# From Interferon to Checkpoint Inhibition Therapy—A Systematic Review of New Immune-Modulating Agents in Bacillus Calmette–Guérin (BCG) Refractory Non-Muscle-Invasive Bladder Cancer (NMIBC)

**DOI:** 10.3390/cancers14030694

**Published:** 2022-01-29

**Authors:** Susanne Deininger, Peter Törzsök, Michael Mitterberger, Maximilian Pallauf, David Oswald, Christian Deininger, Lukas Lusuardi

**Affiliations:** 1 Department of Urology and Andrology, Salzburg University Hospital, Paracelsus Medical University, 5020 Salzburg, Austria; p.toerzsoek@salk.at (P.T.); m.mitterberger@salk.at (M.M.); m.pallauf@salk.at (M.P.); d.oswald@salk.at (D.O.); l.lusuardi@salk.at (L.L.); 2 Comprehensive Cancer Center, Medical University of Vienna, 1090 Vienna, Austria; 3 Department of Orthopedics and Traumatology, Salzburg University Hospital, Paracelsus Medical University, 5020 Salzburg, Austria; c.deininger@salk.at; 4 Spinal Cord Injury and Tissue Regeneration Center Salzburg, Institute of Tendon and Bone Regeneration, Paracelsus Medical University, 5020 Salzburg, Austria

**Keywords:** bladder cancer, checkpoint inhibition therapy, Bacillus Calmette–Guérin, BCG, non muscle invasive, NMIBC, nadofaragene firadenovec, oncolytic viruses

## Abstract

**Simple Summary:**

In Bacillus Calmette–Guérin (BCG) refractory non-muscle-invasive bladder cancer (NMIBC), radical cystectomy is the gold standard. The advent of immune checkpoint inhibitors (CPI) has permanently changed the therapy landscape of bladder cancer (BC). Immune-modulating (IM) therapies can offer a chance for bladder preservation in the treatment of NMIBC. This systematic review includes four full-text articles and four congress abstracts with IM agents in this setting. Durvalumab plus Oportuzumab monatox, Pembrolizumab, and Nadofaragene firadenovec (NF) show complete response (CR) rates of 41.6%, 40.6%, and 59.6% after 3 months. Instillations with oncolytic viruses such as NF and CG0070 show good efficacy without triggering significant immune-mediated systemic adverse events. Recombinant BCG VPM1002BC could prove to be valid as an alternative to BCG in the future. The recombinant pox-viral vector vaccine PANVAC™ is not convincing in combination with BCG. Interleukin mediating therapies, such as ALT-803, are currently being studied.

**Abstract:**

Background: In Bacillus Calmette–Guérin (BCG) refractory non-muscle-invasive bladder cancer (NMIBC), radical cystectomy is the gold standard. The advent of immune checkpoint inhibitors (CPIs) has permanently changed the therapy landscape of bladder cancer (BC). This article presents a systematic review of immune-modulating (IM) therapies (CPIs and others) in BCG-refractory NMIBC. Methods: In total, 406 articles were identified through data bank research in PubMed/Medline, with data cutoff in October 2021. Four full-text articles and four additional congress abstracts were included in the review. Results: Durvalumab plus Oportuzumab monatox, Pembrolizumab, and Nadofaragene firadenovec (NF) show complete response (CR) rates of 41.6%, 40.6%, and 59.6% after 3 months, with a long-lasting effect, especially for NF (12-month CR rate of 30.5%). Instillations with oncolytic viruses such as NF and CG0070 show good efficacy without triggering significant immune-mediated systemic adverse events. Recombinant BCG VPM1002BC could prove to be valid as an alternative to BCG in the future. The recombinant pox-viral vector vaccine PANVAC™ is not convincing in combination with BCG. Interleukin mediating therapies, such as ALT-803, are currently being studied. Conclusion: CPIs and other IM agents now offer an increasing opportunity for bladder-preserving strategies. Studies on different substances are ongoing and will yield new findings.

## 1. Introduction

Bladder cancer (BC) is among the 10 most common cancers worldwide [1]. Smoking is by far the most important risk factor [2]. The tumor histology is mostly of urothelial origin, divergent differentiations, such as squamous, neuroendocrine, micropapillary, sarcomatoid, and others are rarer [3].

In addition to the classification according to TNM [3], BC is subdivided into non-muscle-invasive (NMIBC, up to pT1) and muscle-invasive disease (MIBC, from pT2). Over 80% of tumors are classified as NMIBC at the time of initial diagnosis [4,5].

Tumor grading and the depth of tumor invasion are essential factors influencing the therapy and prognosis. NMIBC is categorized in different risk classes using various systems, predicting the risk for recurrence and progression. Based on the World Health Organization (WHO) 1973 classification system, Sylvester R. J. et al. designed the European Organization for Research and Treatment of Cancer (EORTC) scoring model [4], which includes the following risk factors: number, diameter, grading, and T category of the tumor(s), and concomitant Carcinoma in situ (CIS).

Recently, the European Association of Urology (EAU) updated the scoring model for the disease progression of NMIBC using the newer version of the WHO classification system 2004/2016 and patient data from over 3400 NMIBC cases [6].

In addition to the already mentioned EORTC risk factors, age > 70 years is now an additional independent risk factor.

The one-year risk of progression to muscle invasive disease ranges from 0.06%, to 1.0%, to 3.5% and to 16% for low, intermediate, high-and very high-risk tumors, respectively [7].

The patients’ further therapy is decided based on risk classification. Except for CIS, all forms of NMIBC are primarily resectable by the transurethral resection of bladder tumor (TURBT), regardless of localization and other surgical features [7].

For intermediate-risk patients, the EAU guidelines recommend intravesical chemotherapy (iCT) for up to 12 months or instillation with Bacillus *Calmette–Guérin* (BCG) for 12 months. In the case of previous iCT, BCG therapy for 12 months is indicated. In high-risk NMIBC, BCG bladder instillation therapy should be administered for 1–3 years [7].

Mycobacterium bovis BCG represents a bacterial species discovered by the two researchers A. Calmette and C. Guérin [8]. In the late 1970s and early 1980s, Morales et al. published data from CIS patients treated locally and systemically with BCG. Subsequently, the patients showed a median recurrence-free survival (RFS) of 22.6 months [9,10]. Since then, BCG has become a standard drug in the treatment of intermediate- and high-risk NMIBC.

Introduced by Morales [10], BCG instillation therapy starts with an induction phase of 6 weeks, with instillations once weekly, starting from at least 2 weeks post TURBT. If the patient is subsequently tumor free, a maintenance phase follows, for which a wide variety of schemas exist. One of the best known is certainly the one established by Lamm et al. [11], but many more exist [12,13].

BCG reduces the risk of progression of NMIBC [13] by up to 27%, and the risk of recurrence by 32% compared to bladder instillations with Mitomycin C (MMC).

However, therapy with BCG also has its downsides.

In an intention-to-treat analysis of almost 1400 intermediate-and high-risk NMIBC patients with BCG therapy, Oddens et al. described a recurrence rate of 43.3% over a median FU period of 7.1 years [14].

BCG is not a therapy that is suitable for all groups of patients. As an immunoactive substance, it is not appropriate for immunocompromised persons such as persons with a human immunodeficiency virus (HIV) infection, or persons taking immunosuppressants such as corticosteroids [15,16]. BCG allergies can manifest as rashes or arthralgias [16]. Common side effects are hematuria, dysuria, and urinary tract infections (UTI), while severe adverse events (AEs) such as BCG sepsis or fever are rarely seen [17]. Between 13% and 19% of patients discontinue therapy because of AEs [17,18].

A recent phenomenon is the BCG shortage. The closure of production sites, production downturns, and the discontinuation of a BCG strain produced by Sanofi recently limited access to BCG for some patients [19].

**Figure 1:**
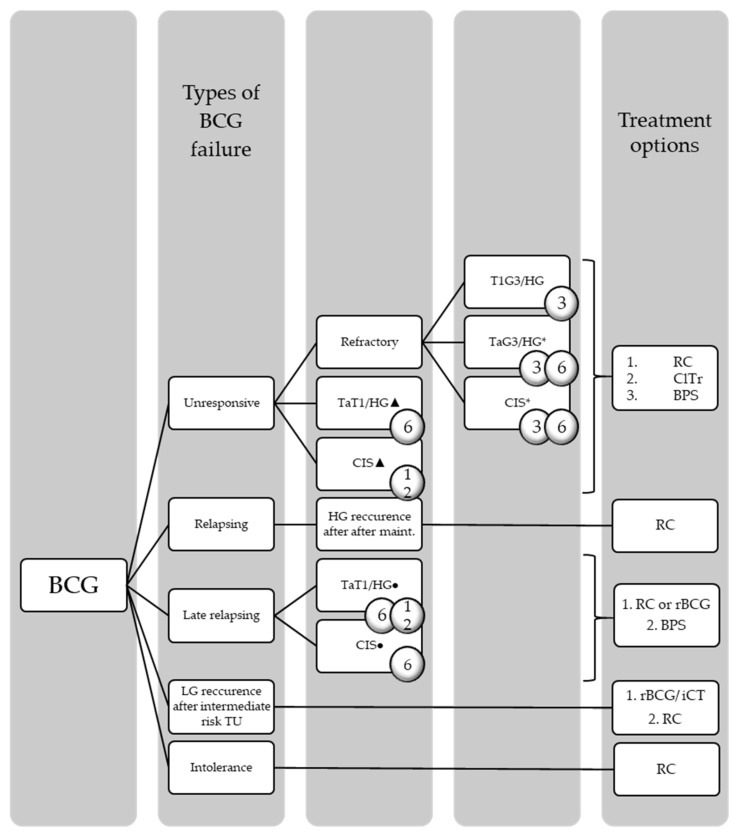
Types of BCG failure and treatment recommendations according to the EAU guidelines on NMIBC = number of months after the initiation of BCG exposure; * after re-induction or maintenance; ▲ = despite adequate BCG exposure (at least 5 of 6 doses of an initial induction course + at least 2/6 doses of a second induction course or 2/3 doses of maintenance; ● = of last BCG exposure; TU = tumor).

When a papillary tumor or CIS progresses to MIBC, there is a clear indication for radical cystectomy (RC) with urinary diversion and pelvic lymph node dissection. However, in BCG failure in high-risk NMIBC, RC is also the treatment of choice. Types of BCG failure and the appropriate treatment recommendations according to the EAU guidelines on NMIBC can be found in Figure 1 [7].

However, RC is a major surgical procedure with numerous possible complications. The median age of RC patients is approximately 70 years [20,21], and many of them have comorbidities or their general condition is reduced. There is thus a demand for a bladder-preserving strategy (BPS), particularly for BCG refractory cases.

Intravesical Valrubicin was approved based on study data from the year 2000, which showed complete response (CR) rates of 21% in this setting [22], though it is not recommended by the international BC guidelines [7]. In addition, there are numerous clinical trials (ClTr) testing chemotherapy, oncolytic viruses, CPI, etc. What they mostly have in common is that the prognosis worsens with the previous number of BCG courses given. In 2020, Kamat et al. published a systematic review and meta-analysis comparing four randomized controlled trials (RCTs) and 24 single-arm studies in BCG refractory NMIBC. The 12-month CR rate was 24% in studies with at least two previous BCG courses, and 36% in studies with at least one BCG course. Nonetheless, the interpretation of the study outcomes is hampered by different definitions of the BCG refractory status within the study population and a lack of information regarding prior BCG therapies [23].

The advent of checkpoint inhibition (CPI) therapies in the treatment of BC has also opened up new opportunities in NMIBC. Initially, programmed cell death ligand-1 (PD-L1)-inhibitors were only used in metastatic (m)BC. The first CPI to be approved by the United States Food and Drug Administration (FDA) for second-line therapy after platinum-based CT and later in platinum-ineligible patients was Atezolizumab (Tecentriq^®^) [24,25,26]. In the same indications, the approval of Pembrolizumab (Keytruda^®^) followed soon after [27,28]. According to data from the JAVELIN Bladder 100 study, the CPI Avelumab (Bavencio^®^) was approved in the maintenance treatment of mBC after platinum-based CT [29].

Nonetheless, other immunotherapeutic drugs are being studied intensively for the treatment of BCG refractory NMIBC. The results are promising. This review addresses new immunomodulatory therapies in this indication.

## 2. Materials and Methods

### 2.1. Literature Search

A literature search was performed by the authors through PubMed/Medline to identify available studies testing immunotherapy in patients with BCG refractory NMIBC. Data cutoff was October 2021. The search was executed using the following search terms in different combinations: “BCG/Bacillus Calmette–Guérin refractory/resistant”; “carcinoma in situ”; “checkpoint inhibitors”; “immunotherapy”; “interferon”; and “NMIBC/non-muscle-invasive bladder cancer”. The reference lists of the studies identified were also used to find additional relevant studies. Current abstracts with preliminary data from international congresses, such as the American Urologic Association (AUA) meeting, the Genitourinary Cancers Symposium of the American Society of Clinical Oncology (ASCO-GU), and the European Association of Urology (EAU), were reviewed regarding the research question.

### 2.2. Study Selection

**Figure 2:**
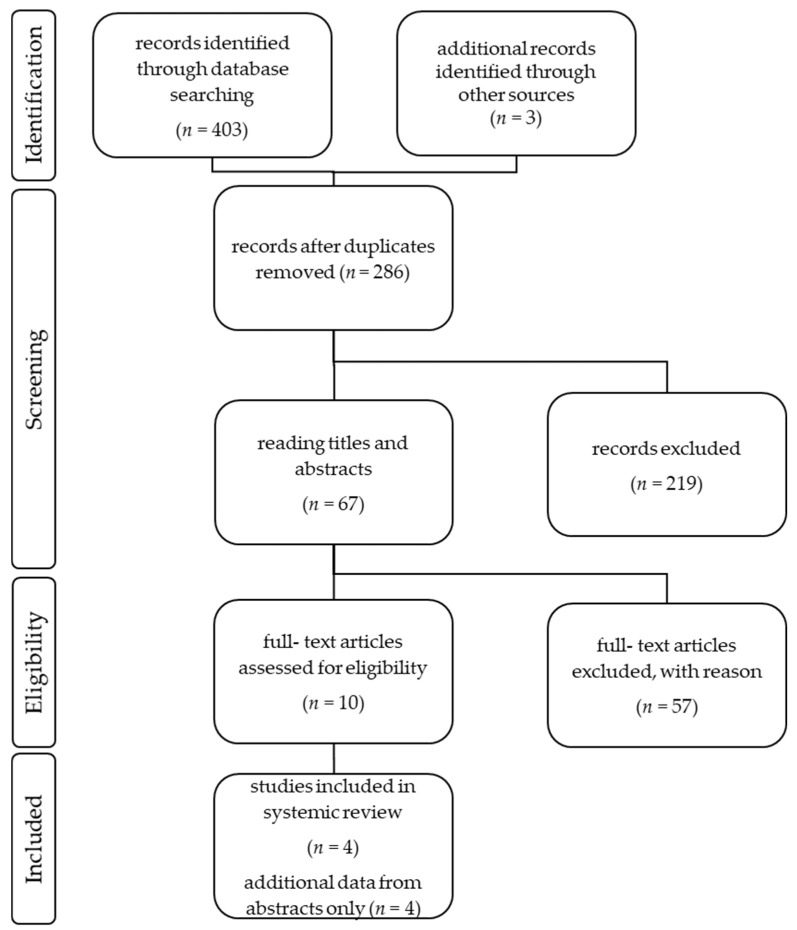
An overview of the study selection following the recommendations of the PRISMA statement.

The authors performed the study selection following the recommendations of the Preferred Reporting Items for Systematic Review and Meta-Analysis Statement (PRISMA) [30]. The study selection process can be seen in Figure 2. A total number of 406 articles was identified through data bank research. Only publications written in English were included. Duplicated studies were excluded. All included publications described prospective, multicenter pharmaceutical studies, with one treatment arm. Case reports or retrospective data analyses were excluded. The abstracts of the publications were checked for eligibility and if the criteria were met, the entire article was reviewed. In total, 10 full-text articles were assessed for eligibility, but *n* = 6 did not meet the research question and were thus excluded. Four full-text articles were included in the review, plus a total number of four congress abstracts.

### 2.3. Cochrane Collaboration’s Tool for Assessing Risk of Bias 

All four full-text articles included [31,32,33,34] reported single-arm studies. Hence, a random sequence generation, allocation concealment, and blinding of participants and personnel was not performed. According to the Cochrane Collaboration’s tool for assessing risk of bias [35], the risk of bias must be considered high. In included abstracts, no information on group assignments or randomizations is available.

### 2.4. Data Extraction

Data extraction was performed by the authors. A uniform data table was used, which contained the following information: general information of the publication (name, authors, journal and year of publication), type of study, study phase, details concerning randomization, baseline characteristics of the patients (number, mean value and standard deviation of age, gender, Eastern Cooperative Oncology Group (ECOG), histology, T-stage, outcome parameters including CR, duration of complete response, stable disease (SD), progressive disease (PD), progression-free survival (PFS), and event-free survival (EFS), and rate of AEs (in ≥10% of patients, and ≥G3 AEs in ≥5% of patients). In all included studies, response rates were determined using response evaluation criteria in solid tumors (RECIST) version 1.1 [36]. The included articles and abstracts are from the years 2018, 2020, and 2021.

### 2.5. Definition of Terms

#### 2.5.1. BCG-Refractory, -Unresponsive, and -Relapsing

The different types of BCG failure and the corresponding therapy options according to the EAU guidelines on NMIBC [15] can be found in Figure 1.

#### 2.5.2. Terms for Response to Therapy

For comparability, the response rates were defined as follows:

CR: no evidence of tumor, or free from HG tumor recurrence;

SD: same stage/grade NMIBC recurrence/persistence;

PD: stage or grade worsening, or progression to muscle-invasive disease, or death from UC or other causes

## 3. Results

### 3.1. CPI

#### 3.1.1. Atezolizumab (Tecentriq©)

The 18-month follow-up (FU) data of the SWOG S1605 clinical trial (NCT02844816) were presented at the 2021 ASCO-GU congress [37,38]. This single-arm phase II trial evaluated the CPI Atezolizumab in patients with BCG-unresponsive high-risk NMIBC who were ineligible for or refused RC. The study drug was intravenously (i.v.) administered at the dosage of 1200 mg every 3 weeks for 1 year. The patients were divided into two cohorts according to the primary T-stage: CIS and TaT1/HG tumors. Treatment success was checked after 3 months by cystoscopy and urine cytology, and after 6 months by cystoscopy, urine cytology, and bladder biopsy. Out of the 172 patients enrolled, 166 received at least 1 dose of the study drug, and were thus included in the safety analysis, whereas *n* = 128 were included in the efficacy analysis [37]. The baseline characteristics of study participants of this and the following clinical studies can be found in Table 1.

From the CIS cohort (*n* = 74), 27% of patients had a 6-month CR, and a median duration of response (DoR) of CR of 15.4 months. The CR rates of this and the following clinical studies divided according to their initial T-stage can be found in Table 2.

The 18-month EFS was 47.1%, 17%, and 29%, in the TaT1/HG cohort (*n* = 55), the CIS cohort, and in the overall population, respectively. Three patients progressed to muscle-invasive disease at 3 (*n* = 2) and at 22 months (*n* = 1). Two patients developed metastatic disease without bladder recurrence at 17 and 31 months. The data on the patients who had to undergo RC are still being evaluated.

The rate of treatment-related AEs was 85.5% in the safety cohort (*n* = 166), with 16.9% ≥ G3 treatment-related AEs. The general rate of treatment-related AEs of this and the following clinical studies can be found in Table 3. The most common AEs were fatigue in 43.4%, anemia in 22.9%, and diarrhea in 20.5%. No specific ≥G3 AE was experienced by ≥5% of patients.

Atezolizumab is also currently being investigated in combination with BCG in BCG-naïve high-risk NMIBC as part of the phase 1 BladderGATE trial (NCT04134000) [47].

#### 3.1.2. Pembrolizumab (Keytruda©)

Data on the Keynote-057 trial were published in July 2021 [32]. The trial investigated Pembrolizumab in patients with BCG-refractory, high-risk NMIBC who were ineligible for or refused RC. The patients received 200 mg Pembrolizumab i.v. every 3 weeks for up to 2 years. Patients were divided into two cohorts according to the primary T-stage: CIS +/−TaT1/HG tumors (cohort A) and TaT1/HG tumors only (cohort B). The publication contains data from cohort A only. Treatment success was checked every 3 months by cystoscopy and urine cytology, by biopsies in the case of abnormal findings, and by computerized tomography urogram every 6 months for the first 2 years.

Out of the 101 patients included in cohort A, all received at least one dose of the study drug, and were thus included in the safety analysis, whereas *n* = 96 were included in the efficacy analysis. The patients had a median age of 73 years [48,49,50,51,52,53,54,55,56,57,58,59,60,61,62,63,64] and had received a median of 12.0 (9.0–16.5) BCG instillations. Two patients had received prior systemic therapy. Among the patients, 63.4% had CIS only, whereas 24.9% had CIS + Ta/HG tumor and 11.9% had T1 + CIS tumor. The reason for not undergoing RC was refusal in 95.1% and ineligibility in 2.9%. The BCG failure category was defined as disease recurrence in 69.3% and persistence in 25.7%.

At 3 months FU, for *n* = 96 in the complete cohort, CR was 40.6%, SD was 47.9% (with 41.7% persistence and 6.3% recurrence of disease), and PD was 9.4% (with 9.4% stage or grade worsening, and 0% progression to muscle-invasive disease/metastatic disease/death from any cause). The 3-month CR in the CIS, Ta + CIS, and T1 + CIS cohorts were 45.0%, 29.2%, and 41.7%, respectively. The median OS of the complete cohort was not reached at the time of data cutoff. The 12-month OS was 98%, and 36-month OS was 91%. The 12-month PFS to grade- or stage-worsening or death was 83%. At data cutoff, *n* = 9 patients had died (*n* = 3 deaths due to non-study drug-related AEs, *n* = 3 cause unknown, *n* = 1 senile atrophy, *n* = 1 PD, and *n* = 1 cardioembolic stroke). Pembrolizumab was given over a median time range of 4.2 months with a median number of 7 (5–14) applications. Among the patients, 91.1% (*n* = 101) discontinued treatment, with disease persistence (38.6%) or recurrence or stage progression (33.7%) being the main reasons for discontinuation.

Among the patients in the safety cohort, 66.3% (*n* = 101) experienced some kind of treatment-related AE, with 12.9% ≥ G3 AEs. The most common treatment-related AEs were fatigue, pruritus, and diarrhea (each in 10.9%). No specific ≥ G3 AE was experienced by ≥5% of patients. Among the patients, 12.9% and 6.9% interrupted and discontinued treatment due to AEs, respectively. Immune-related (ir) AEs were reported in 21.8% of patients, with 2.9% ≥G3 irAEs. Seven patients required systemic corticosteroids (6.9%).

In January 2020, Pembrolizumab was FDA-approved for the indication of patients with BCG refractory CIS with or without a papillary tumor that are ineligible for or refuse RC [65]. Currently running is the phase 3 study MK-3475-676/KEYNOTE-676, which investigates Pembrolizumab in combination with BCG BCG naïve patients on the one hand and in BCG refractory high-risk NMIBC patients on the other hand.

#### 3.1.3. Durvalumab (Imfinzi©) in Combination with Vicineum™ (Oportuzumab monatox, OM)

Vicineum™ is a recombinant fusion protein of anti-EpCAM and a truncated form of Pseudomonas Exotoxin A. EpCAM positive BC shows higher grading and staging, and worse OS compared to EpCAM negative tumors [43]. The preliminary published data from the phase 3 VISTA trial had shown convincing data after Vicineum™ mono instillation in BCG-unresponsive NMIBC: the 3-month CR rate was 42% in CIS, and the RFS in TaT1 was 68%. The maximum DoR was 273 days [42]. FDA approval was denied in August 2021 [44], and the application for EMA approval was withdrawn [66].

At the AUA meeting in 2021, the first data from a phase 1 clinical study of Vicineum™ in combination with Durvalumab i.v. in patients with BCG unresponsive NMIBC were presented (NCT03258593). The patients received 30 mg Vicineum™ intravesically weekly for 12 weeks (induction), followed by every week for up to 52 weeks (maintenance) plus Durvalumab 1500 mg i.v. monthly for up to 52 weeks. A total of 12 patients were enrolled, among which 41.7% had CR at 3 months, 33.3% at 6 months, and 16.7% at 12 months.

All patients showed at least one treatment-related AE, with 8.0% ≥ G3 AEs. The most common urinary tract-associated AEs were: hematuria, other renal or urinary disorders, UTI, and urinary frequency (each in 41.7%), bladder spasm, dysuria, micturition urgency, proteinuria, and renal dysfunction (each in 16.7%). Pruritus (33.3%), diarrhea (25.0%), rash (25.0%), maculopapular rash (25.0%), and chills, fatigue, headache, hypotension, insomnia, increased amylase, hyperthyroidism, and dry skin (each 16.7%) were the most common systemic AEs. The most common ≥ G3 AEs were hyponatremia and thromboembolism (both 8.3%).

At weeks 3–5, urinary biomarker analyses showed a decrease in EpCAM excretion and a 5–10 fold increase in PD-L1 [39].

### 3.2. Virus Vector-Mediated Immunotherapy/Immunostimulation and Vaccines

#### 3.2.1. The Oncolytic Virus CG0070: Replication in Retinoblastoma (Rb)-Pathway Deficient UC Cells and Immune Induction via Granulocyte-Macrophage Colony-Stimulating Factor (GM-CSF)

CG0070 represents an oncolytic adenovirus, which replicates in Rb-deficient UC cells. In 2012, data from the phase 1 BOND study investigating CG0070 in patients with NMIBC unresponsive to at least one intravesical therapy were published [40]. This study showed a CR rate of 48.6% across all dose expansion cohorts and a median DoR of 10.4 months. In patients with borderline or high Rb phosphorylation, the CR rate was >80%. The researchers assumed the replication of the virus in BC cells because of increasing viral genomes in urine after instillation. In addition, there was a presumptive immune induction. In 94.3%, GM-CSF peaked in urine at day 2 post-instillation, and in 84.6%, GM-CSF was transiently detectable in blood between days 2 and 8 post-instillation.

In the subsequent BONDII study [31], patients received a fixed dose of 10^12^ viral particles (Vp) intravesically via a transurethral catheter. The solution was induced after pre-treatment with dodecyl maltoside (DDM), an agent used to enhance the transduction efficiency of CG0070. CG0070 was left in situ for 45–50 min, and the patients were encouraged to move around. The instillations performed were similar to those for BCG: once weekly for 6 weeks (induction) followed by once weekly for 6 weeks every three months (maintenance). The success of the therapy was checked by cystoscopy and urine cytology after 3 months, and mandatory bladder biopsies after 6 months.

In this study, response to therapy was defined as follows:

CR: negative cystoscopy, two consecutive negative urine cytologies;

PD: any evidence of disease after prior negative assessment, or progression to ≥T2 stage.

Low-grade papillary tumor recurrence was not classified as local recurrence and was consecutively resected. Six patients were available for 3-month efficacy analysis, but efficacy data are only shown for *n* = 45 patients available for 6-month FU.

In the CIS cohort, the 6-month CR was 58.3%, SD was 12.5%, and PD was 29.2%. Six-month response rates were the following for the Ta + CIS cohort: CR in 37.5%, SD in 50.0%, and PD in 12.5%. For the T1 + CIS cohort, 6-month CR was 25.0%, SD was 50.0%, and PD was 25.0%. For the TaT1, only the cohort CR, SD, and PD were equally 33.3% after 6 months. Three patients died during the study: one patient from a cardiac event; one patient from esophageal cancer; and one patient from MIBC.

The rate of AEs was evaluated in 67 patients. Among them, 56.7% experienced at least one treatment-related AE. The most common urinary tract-associated AEs were: bladder spasms in 35.8%; hematuria in 32.8%; urinary tract pain in 29.9%; urgency in 19.4%; UTI in 16.4%; and pollakiuria in 14.9%. Fatigue (11.9%) and flu-like symptoms (10.4%) were the most common systemic AEs.

CG Oncology announced in early September 2021 the planning of a study with CG0070 in combination with Nivolumab in metastatic BC [41].

#### 3.2.2. Nadofaragene Firadenovec (rAd-IFNa/Syn3, Instiladrin^®^): Immune Enhancement via IFNα

Nadofaragene firadenovec is a non-replicating adenovirus rAd-IFNα that transfers the human Interferon (IFN) α-2b gene into urothelial cells. The adenovirus is coupled with Syn3, a polyamide surfactant that enhances gene transfer and expression [45]. This triggers a local release of IFN α-2b. The drug is intravesically applied. In a phase 1 study, high and long-lasting IFNα levels were measurable in the urine after the application of rAd-IFNa/Syn3, and the therapy was well tolerated [46].

A phase 2 study was then conducted in which 43 patients with BCG unresponsive NMIBC (TaT1/HG+/−CIS, or CIS only) intravesically received 1 × 10^11^ Vp/mL or 3 × 10^11^ Vp/mL [67]. HGRFS was 23%, 17%, 17%, and 14% after 3, 6, 9, and 12 months for both dosages, respectively. Urine from all patients showed significant IFNα-2b levels at days 2, 4, and 12 after the first instillation, and at days 2 and 4 after the second instillation (if performed). No clinical correlation of IFNα-2b levels and dosage or response was detected.

Data from the subsequent phase 3 study was published in 2021 [33]. Patients received a single bladder instillation with 3 × 10^11^ Vp/mL of Nadofaragene firadenovec on months 0, 3, 6, and 9. The instillation was left in situ for 1 h and the patients were encouraged to move around.

The success of the therapy was checked by cystoscopy and urine cytology every 3 months, and by mapping biopsies at 12 months. The instillations could be continued every 3 months in patients with CR and with the consensus of the practitioner and patient.

A total of 157 patients received at least 1 dose of the study drug, but 6 patients did not meet the criteria for BCG refractory NMIBC, hence *n* = 151 were included in the efficacy analysis. In the CIS only cohort, CR was 53.4% at 3 months, 40.8% at 6 months, 35.0% at 9 months, and 24.3% 12 months. In this cohort, the median DoR was 9.7 months. In the TaT1/HG cohort, CR was 72.9% at 3 months, 62.5% at 6 months, 58.3% at 9 months, and 43.8% 12 months. The median DoR was 12.4 months. In the complete cohort, CR was 59.6% at 3 months, 47.7% at 6 months, 42.4% at 9 months, and 30.5% at 12 months. The median DoR was 7.3 months. Among the patient, 26% had sought RC by data cutoff at 12 months: 29.1% of patients out of the CIS cohort, and 20.8% of patients out of the TaT1/HG cohort. Five patients died during FU: *n* = 3 due to cardiac event; *n* = 1 due to pneumonia; and *n* = 1 due to an unknown cause.

Among the patients, 157 were included in the safety analysis, among which 70.1% experienced some kind of treatment-related AE, with 3.8% ≥ G3 AEs. The rate of treatment-related sAEs was 1.9%, and the rate of study drug discontinuation because of AEs was 1.9%. There were no G5 AEs. The most common urinary tract-associated AEs were: discharge around the catheter in 24.8%, bladder spasm in 15.9%, micturition urgency in 15.3%, and dysuria in 10.8%. Fatigue (19.7%), chills (11.5%), and fever (10.2%) were the most common systemic AEs. No specific ≥ G3 AE was experienced by ≥5% of patients.

The FDA approval of Nadofaragene firadenovec is still pending at the time of data cutoff of this article.

#### 3.2.3. Recombinant Pox-Viral Vector Vaccine PANVAC™ Plus BCG

PANVAC™ contains two viral vectors: vaccinia and recombinant fowlpox. The vectors contain transgenes for the two tumor-associated antigens epithelial mucin 1 (MUC1) and carcinoembryonic antigen (CEA), as well as for three costimulatory molecules of human T cells (B7.1, intracellular adhesion molecule-1 and leukocyte function-associated antigen-3). PANVAC™ has been shown to enhance CEA and MUC1-specific cytotoxic T-lymphocyte response [68] in preclinical models and has already been tested in diverse tumor entities including pancreatic [69] and colorectal cancer [70].

At the 2021 AUA Meeting, the first data from a phase II study testing the combination PANVAC™ with BCG vs. BCG alone (1:1) in patients with NMIBC who failed at least one course of BCG were presented [71,72].

During the study, BCG was administered in six weekly instillations, and the study drug PANVAC™ was given in five injections over a time period of 15 weeks [73].

A total of 30 patients were included. Initial T-stage was as follows: CIS alone in 20.0%, Ta + CIS in 6.7%, T1 + CIS 3.3%, Ta in 46.7%, and T1 23.3%. At 12 months, the RFS was equal between both cohorts (42.9% in the BCG alone cohort, and 44.4% in the BCG + PANVAC™ cohort, *p* = 0.97). The median time to recurrence likewise showed no difference (11.7 vs. 9.9 months, *p* = 0.77). Twelve-month PFS was 79.6% for BCG alone, and 78.6% for PANVAC™ + BCG [74]. Among the patients, 30% sought RC by data cutoff at 12 months.

Because of the fact that only preliminary data are available for this clinical study, the data were not included in Table 2 and Table 3.

### 3.3. Modified BCG

#### VPM1002BC

VPM1002BC is a recombinant Mycobacterium bovis BCG. Here, the listeriolysin gene of Listeria monocytogenes is inserted into the Urease-C gene. Thus, Urease-C is inhibited, leading to the acidification of the environment in the host phagosome. In the acidic pH, listeriolysin is then activated, which perforates the host’s membrane. The researchers assumed that immune activation is at least comparable to conventional BCG with better tolerability [34]. Data on the SAKK 06/14 study were published in 2020. In this phase I/II trial, six patients with NMIBC unresponsive to BCG with a median age of 74 (71–82) years were enrolled. All had an ECOG 0. The initial T-stage was CIS, T1, and Ta in 50.0%, 33.3%, and 16.7%, respectively.

Similarly to BCG, the patients received six weekly instillations of VPM1002BC (induction), followed by three weekly instillations at 3, 6, and 12 months (maintenance). The results showed increased plasma levels of TNFα as well as increased GM-CSF and IFN expression in CD4+ T-cells. No ≥ G3 AEs were recorded, and no dose limiting toxicity was documented.

Since the study is a phase I/II study with a small patient collective, the data were not included in the figures and tables.

### 3.4. IL Agonists

#### IL-15RαFc Superagonist ALT-803 Instillation Plus BCG

ALT-/N-803 (Anktiva^®^) is an IL-15RαFc superagonist which stimulates the proliferation and activation of NK cells and CD8+ T cells via L-2 and 15βγ receptor, without upregulating T-regulatory cells [75]. In a phase 1 study, ALT-803 instillations were evaluated in combination with BCG in nine patients with high-risk NMIBC, resulting in a 24-month CR of 100%.

The first data from the following phase 2/3 study QUILT 3.032 were presented at the e2021 ASCO-GU congress [76]. By data cutoff in December 2020, *n* = 80 patients with CIS+/−TaT1/HG tumors refractory to BCG therapy were included. All patients received instillations of BCG + 400 µg ALT-803 according to standard BCG schemes (3× induction plus 3× maintenance).

At the time of data cutoff, 71.8% achieved CR, with a median duration of CR of 19.2 [7.6–26.4] months.

The most common AEs were urinary tract associated: dysuria (16%), urinary urgency (14%), and bladder spasms (8%). No therapy-associated irAEs, sAEs, or G5 AEs occurred. No treatment was interrupted because of AEs. At the time of data cutoff, 12.5% of patients sought RC.

Since only preliminary data were available for this clinical study, it was not included in Table 2 and Table 3.

This one and other ongoing/upcoming studies on immune-modulating therapies in BCG refractory NMIBC can be found in Table 4.

## 4. Discussion

The CUETO and the EORTC showed the influence of clinical factors such as age, recurrence rates, and sex on the response of NMIBC to BCG instillation therapy [4,5]. The precise molecular mechanisms are still poorly understood. To explain the processes concerning the immune system in BC, the natural immune system in the healthy urothelium must first be understood. Many mechanisms are still not clear, as few studies exist about the immune landscape in the urinary bladder. CD4+ positive T-lymphocytes and macrophages seem to be present in mice and human urothelium [77]. CD4+ cells can differentiate into various T-helper cells or regulatory T-cells in response to certain stimuli [78]. In the urinary bladder of the mouse, 70% of CD45+ cells have been shown to be antigen-presenting cells (APCs), among which 40% were macrophages and 20% were dendritic cells. The rest was composed of CD4 T-lymphocytes, γδ-T lymphocytes, and other mast cells and natural killer T-(NKT-) cells [79]. In addition to resident bladder immune cells, circulating immune cells can be recruited. Tumor formation, however, triggers a complete change in the natural immune cell environment of the urothelium. The BC-associated release of specific factors causes the influx of neutrophils, forkhead box protein P3 (FoxP3) + regulatory T-cells and myeloid-derived suppressor cells (MDSCs) [77]. Both FoxP3+ cells (TILs and BC cells) and MDSCs seem to have prognostic significance in BC and other tumor entities [48,49].

MDSCs assist in the construction of an immunosuppressive tumor microenvironment and are involved in tumor metastasis and angiogenesis [49].

In recent years, there has been an increasing amount of data indicating that some tumor entities are more heavily loaded with lymphocytes than others, which seems to play a crucial role in the prognosis of disease. In lung cancer, for example, high levels of CD8+ (=cytotoxic) tumor-infiltrating lymphocytes (TILs) seem to have a positive prognostic effect, whereas high levels of FoxP3+ regulating TILs are associated with a poorer prognosis [50]. The same is true for colorectal cancer [51]. In NMIBC, data show a correlation between the number of CD4+ and CD8+ TILs and a higher risk of recurrence and poor prognosis [52,53].

Immunotherapy in BC therapy has a long tradition: BCG has been successfully used for over 30 years [18], even though its mechanism has not yet been fully elucidated. On the one hand, a direct cytotoxic effect on BC cell lines is suspected: the induction of apoptosis via the Toll-like receptor (TLR) 7 [54] and of necrosis via direct cell wall damage [55] has been shown in vitro. In addition, the cause of oxidative stress via the release of reactive oxygen species such as H_2_O_2_ is also suspected to be directly cytotoxic [56]. On the other hand, BCG also seems to act indirectly via immune induction. After uptake via the phagocytosis endocytosis mechanism into the BC cells [57], the activation of the TLR on BC cells and APC leads to the activation of NF-κB, which triggers the release of immune-activating cytokines in the cell nucleus [58]. The CD4+ T-lymphocytes secrete cytokines such as IL-1/2/6/8/10/12/17/18, TNF-α, IFN-γ, and GM-CSF, which in turn activate CD8+ cytotoxic T-cells, macrophages, neutrophils, and NKT-cells, or directly kill the BC cells such as IFN-γ [59]. Moreover, BC cells, after internalizing BCG, appear to become APCs themselves. These and other APCs process and present antigens via major histocompatibility complex class II (MHC-II) molecules, which leads to a response of CD4+ and CD8+ T-lymphocytes [60].

VPM1002BC is supposed to work similarly to recombinant Mycobacterium bovis BCG. Compared to BCG, MHC-mediated antigen binding after the internalization of VPM1002BC leads to an increased CD4+ and CD8+ T-cell response in the mouse model, as well as an increased concentration of IL-6/10/23 [61]. VPM1002BC also seems to activate BC apoptosis and pyroptosis stronger compared to standard BCG by activating different caspase pathways [62,63,64]. VPM1002BC still has to prove its efficacy and safety in phase II and III studies compared to the well-established BCG. It may eventually represent an alternative approach to the classical BCG therapy in the future. However, it is uncertain whether it can take a place in the therapy of patients with BCG failure, especially since it targets the same pathways.

There is evidence that BCG failure is due to the expression of alternative immune pathways, e.g., PD-L1. Even more, PD-L1 seems to be a predictor of BCG response, and in some studies, PD-L1 expression changes with BCG therapy.

Lim et al. examined the blood and tissue samples of BCG-treated patients in 2020. BCG responders’ tissue samples were enriched with active CD8+ PD-1 negative TILs, whereas non-responders’ samples showed increased exhausted CD8+ PD-1+ TILs [80].

Kates et al. came to a similar conclusion in 2020: the baseline (before BCG) PD-L1 expression of BCG non-responders was significantly higher compared to BCG responders (*p* < 0.01) [81], with no difference in CD4+, CD8+, or FoxP3 between the two cohorts. BCG therapy did not increase PD-L1 gene expression. Controversially, Wang et al. showed in vitro and in vivo that BCG can increase the PD-L1 expression of BC cells via the mitogen-activated protein kinase (MAPK) pathway, which may lead to a reduced effect of cytotoxic T-lymphocytes [82,83]. In addition, the combination of BCG with a CPI in the rat model led to an increased activity of CD8+ T-lymphocytes, as well as to a reduction in MDSCs [83]. These data may suggest that CPI may provide a particularly good starting point in the treatment of BCG refractory NMIBC.

Nonetheless, all patients included in the Keynote-057 trial were BCG non-responders, but not all of them had high PD-L1 expression and thus a high combined positive score (CPS). The CPS score was the number of PD-L1 positive cells divided by the number of PD⁠-L1 positive and negative tumor cells, multiplied by 100 [84]. Among them, 38% had a CPS ≥ 10, while 57% were <10 [42]. In addition, PD-L1 expression was not a predictor of response in this study. This finding is contradicts the preclinical data and needs further investigation.

The combination of PD-L1 inhibitors with EBRT may also be a promising approach and is currently being investigated in the PREVERT trial (NCT03950362). Radiation also has an immunomodulatory effect via ILs and APCs [85], and shows effects in irradiated and non-irradiated lesions in several tumor entities in preclinical models (compare abscopal effect) [86]. This and other ongoing/upcoming studies on the topic of immune-modulating therapies in BCG-refractory NMIBC can be found in Table 4.

The relationship between EpCAM and PD-L1 is still unclear. However, treatment with EpCAM antibodies seems to decrease PD-L1 protein levels and increase the activity of cytotoxic CD8+ TILs in vitro and in mouse tumor models. The combination of Atezolizumab and the EpCAM antibody shows a stronger CD8+ immune response compared to CPI therapy alone [87]. The combination of EpCAM antibodies such as Vicineum^®^ with CPI may be an interesting approach, as BCG-refractory NMIBC tends to have a high EpCAM expression [88].

Another immunomodulatory approach, which has been studied for years in the treatment of NMIBC, is the administration of IFNα. The agent has been used in the treatment of diseases such as hepatitis C and in oncological tumor entities such as lymphoma, leukemia and melanoma for decades [89]. Since the advent of tyrosine kinase inhibitors, and later CPIs, it has already lost its place in the treatment of renal cell carcinoma. Unlike INFγ mentioned above, which is a Type II IFN, IFNα is a family of Type I IFNs that attracts NKT-cells, promotes MHCI response and enhances antibody recognition [90]. The combination of BCG and IFN has been studied in the treatment of high-risk NMIBC. However, a meta-analysis of four RCTs showed no benefit for BCG plus IFNα compared to BCG alone in terms of recurrence (risk ratio (RR) 0.76), progression (RR 0.26), or disease-specific mortality (RR 0.38). When alternating with IFNα, BCG even resulted in a shorter time-to-recurrence for the combination compared to BCG alone (hazard ratio 2.86) [89]. Given as monotherapy, however, IFNα bladder instillations showed CR in 43% of patients with CIS in one RCT [91]. Unfortunately, the success of the therapy did not last long, as the authors suspected, due to the short duration of the exposure of IFNα in the urinary bladder of 1–2 h [33,91]. Hence, another formulation of the drug had to be found. The adenovirus vector-based gene therapy via rAd-IFNa/Syn3 enabled the induction of local IFN exposure for days while minimizing potential systemic side effects. In the rat model, after rAd-IFNa/Syn3 instillation into the urinary bladder, IFNα-2b release via urine was evident, whereas the systemic release of IFNα-2b was minimal [92].

The aforementioned 2021 clinical study testing rAd-IFNa/Syn3 showed good response rates, especially in the cohort with papillary tumors (3-and 6- months CRs 72.9% and 62.5%) ahead of the group of patients with CIS (3- and 6-month CRs of 53.4% and 40.8%, respectively) [33]. Even though the response rates of papillary tumors were not individually reported for other therapies except for CG0070 (6-month CR rate of 33.3%), the 3- and 6-month CR rate for rAd-IFNa/Syn3 is better than that for all other therapies and T-stages. Moreover, the effect of rAd-IFNa/Syn3 seems to be long-lasting: even after 12 months, a CR is still present in 30.5% of the complete study cohort, compared to 16.7% with Durvalumab + OM and 9.4% with Pembrolizumab. The rate of severe AEs also seems to be better in rAd-IFNa/Syn3 compared to CPI and CPI combination therapies: only 3.8% of patients had ≥G3 AEs compared to 8.0%, 12.9%, and 16.9% for Durvalumab + OM, Pembrolizumab, and Atezolizumab, respectively. The clinical study also confirmed the preclinical data of the mainly local effect: most AEs were urinary tract associated.

CG0070, as another virus vector-mediated therapy, exerts its immune effect via the stimulation of GM-CSF release. In vitro and in vivo studies performed by Dranoff et al. in 2002 showed a long-lasting immune response of CD4+ and CD8+ T cells, NKT-cells and antibodies after vaccination with GM-CSF secreting irradiated tumor cells [93]. Subsequently, intravesical GM-CSF application showed a reduction in the incidence of bladder tumors in the mouse model by 72.5 percentage points, as well as an induction of resistance to tumor recurrence [94]. An interesting approach is the combination of CG0070 with CPI, as the combination of GM-CSF with CT (in this case gemcitabine) and CPI was shown to prolong local recurrence-free and cancer-specific survival after RC in a small study population [95]. Furthermore, in T-cell lymphoma, there are data indicating that GM-CSF promotes immune activation via the upregulation of PD-L1 expression in NKT-cells [96]. In May 2020, the CORE-001 clinical trial started to investigate the instillation of CG0070 with CPI therapy with Pembrolizumab (NCT04387461). Data are awaited.

ILs can also be the target of antitumor therapy. IL-10, which is secreted by BC cells [77], leads to polarization and thus to an effect reversal on macrophages: normally anti-neoplastic, polarized M2 tumor-associated macrophages (TAMs) are pro-angiogenic and thus tumor-promoting [97]. A high density of TAMs also seems to have a prognostic significance in NMIBC: RFS worsens with an increasing number of TAMs under BCG therapy [98]. In addition, M2 TAMs secrete fewer cytokines compared to naïve macrophages, which normally attract anti-tumor T-lymphocytes [99]. IL-10, on the other hand, seems to induce PD-L1 expression on the TAMs [100]. IL-2 appears to be a marker for response to BCG therapy in NMIBC [101], and ALT-801, a T-cell-receptor-IL-2 fusion molecule, is currently under investigation in BCG refractory NMIBC in combination with Gemcitabine i.v. in a phase 1b clinical trial (NCT01625260) [102]. ALT-803, an IL-15RαFc superagonist, is tested in combination with BCG in the phase 2/3 QUILT-3.032 trial (NCT03022825, please find data above), and the first data were already published at the 2021 ASCO-GU congress.

## 5. Conclusions

The urinary bladder is an immunogenic organ. The occurrence of BC permanently alters the immune landscape of the bladder. The mechanisms are still poorly understood. BCG has been the only established immunotherapy for high-risk NMIBC for decades. After BCG failure, RC has been the gold standard ever since. It is currently assumed that the development of BCG resistance is based on the development of alternative immune pathways, e.g., from PD-L1. Immunizing therapies, either as bladder instillation or as intravenously or orally administered therapies, may offer a chance for bladder-preserving treatment. The price is low for instillation therapies, as mainly local AEs occur. Systemic therapies, however, rarely cause severe immune-mediated AEs. Ultimately, we need more research, including basic research on the complex interactions of the urinary bladder immune system, in order to offer patients a well-established alternative to RC and not to compromise oncological outcome.

## 6. Limitations

The available number of studies on the topic is small. Many data, especially phase 3 studies, are still in their early phases. In particular, data from the abstracts mentioned may still change relevantly at the time of final publication.

In addition, the studies have defined the response rates differently: CR was defined as “free of high-grade recurrence” in some trials but “free from tumor” in others. In some studies, PD was defined as stage or grade progression or progression to muscle-invasive disease, whilst in others it was defined as tumor recurrence after initial response or death from any cause. In some studies, a response criterion is “free from HG recurrence”, whilst in others it is not. For better comparability, the response criteria CR, SD, and PD were applied to all studies. If mentioned in the original article/abstract, the exact criteria were specified in the corresponding text section. However, this may lead to inaccuracy in the presentation.

In some studies, it is unclear how the criterion BCG refractory (or “unresponsive”) is defined. This is especially the case in the study with PANVAC™ + BCG patients which are unresponsive to at least one course of BCG [73]. This does not meet the EAU guidelines’ on NMIBC [7] definition for BCG refractory. Data on the previous number of BCG instillations and the type of BCG failure are rarely given, and they are inconsistent. Particularly in the preliminary data from congress abstracts, no inclusion criteria regarding the type of BCG failure are mentioned. This leads to the reduced comparability of the studies.

## Figures and Tables

**Table 1:** Baseline characteristics of study participants (* received at least one of the study medications; *Italics*: preliminary data; ^1^ included in efficacy analysis; OM = Oportuzumab monatox).

Medication			*Atezolizumab* [39,40]	*Durvalumab* *+ OM* [41]	Pembrolizumab [42]	CG0070 [43]	Nadofaragene Firadenovec [44]	*PANVAC + BCG* vs. *BCG Alone* [45]		ALT-803 + BCG [46]
								*PANVAC + BCG*	*BCG Alone*	
**Study phase**			*2*	*1*	**2**	**2**	**3**	*1b/2*		*2/3*
Number of patients (*n*)			*128 ^1^*	*12*	101 *	67	157	*15*	*15*	*80*
Median age in years (range)			*NA*	*69.5 (57–82)*	73 (63–79)	72 (64–80)	71 (66–77)	*NA*		*72.5*
Sex (male), %			*NA*	*91.7*	84.2	80.6	82.2	*NA*		*86*
ECOG		0, % ≥1, %	*NA*	*NA*	73.3	NA	89.2	*NA*		*82*
			*NA*	*NA*	26.7	NA	10.8	*NA*		*18*
Median number of previous BCG instillations (range)			*NA*	*NA*	12.0 (9.0–16.5)	NA	NA	*NA*		*16.2*
Initial T-stage	CIS		*74*	*NA*	63.4	46.3	51.6	*6*		*69*
	Ta/HG		*NA*	*50.0*	NA	16.4	22.3	*14*		*0*
	Ta/HG + CIS		*NA*	*NA*	24.9	14.9	13.4	*2*		*21*
	T1		*NA*	*16.7*	NA	11.9	9.6	*7*		*0*
	T1 + CIS		*NA*	*NA*	11.9	8.9	3.2	*1*		*9*
	TaT1/HG		*55*	*NA*	NA	28.4	NA	*NA*		*0*
	TaT1/HG + CIS		*0*	*33.3*	36.8	23.8	16.6	*3*		*30*

**Table 2:** Rate of CR in percentage terms according to initial T-stage (NA = not available).

Initial T-Stage	FU in Months	*Atezolizumab* [39,40]	*Durvalumab* *+ OM* [41]	Pembrolizumab [42]	CG0070 [43]	Nadofaragene Firadenovec [44]
**CIS**	3	*NA*	*NA*	45.0	NA	53.4
	6	*27.0*	*NA*	NA	58.3	40.8
	12	*NA*	*NA*	NA	NA	24.3
**Ta + CIS**	3	*NA*	*NA*	29.2	NA	NA
	6	*NA*	*NA*	NA	37.5	NA
	12	*NA*	*NA*	NA	NA	NA
**T1 + CIS**	3	*NA*	*NA*	41.7	NA	NA
	6	*NA*	*NA*	NA	25.0	NA
	12	*NA*	*NA*	NA	NA	NA
**Complete cohort**	3	*NA*	*41.6*	40.6	NA	59.6
	6	*NA*	*33.3*	NA	NA	47.7
	12	*NA*	*16.7*	NA	NA	30.5

**Table 3:** General treatment-related adverse events (AEs) presented in percentage terms, classified according to the Common Terminology Criteria for Adverse Events (CTCAE) in all included studies (*Italics*: preliminary data; NA = not available).

Medication	*Atezolizumab* [39,40]	*Durvalumab* *+ OM* [41]	Pembrolizumab [42]	CG0070 [43]	Nadofaragene Firadenovec [44]
Number of patients (*n*)	166	12	101	67	157
Treatment-related AE, %	85.5	100.0	66.3	56.7	70.1
Treatment-related ≥ G3 AE, %	16.9	8.0	12.9	NA	3.8

**Table 4:** Ongoing/upcoming clinical trials testing immunotherapy in BCG refractory BC alone or in combination (recruitment status: NYR: not yet recruiting, ANR: active, not recruiting; R: recruiting; EBRT-B: external beam radiotherapy of the bladder; Q3/6W: 3-/6-weekly; * = Indolamin-2,3-Dioxygenase (IDO) inhibitor; Inst: instillations; ♦ = second treatment arm: BCG naïve patients).

Trial Name	PREVERT	ADAPT-BLADDER	Check-Mate 9UT	MK-3475-676/ KEYNOTE-676	CORE-001	QUILT-3.032	ALT-801 in Patients with BCG NMIBC
**NCT number**	NCT03950362	NCT03317158	NCT03519256	**NCT03711032**	**NCT04387461**	NCT03022825	NCT01625260
**Immune mediating agent**	Avelumab 10 mg/kg i.v. Q3W for 8 cycles	Durvalumab 1120 mg i.v. Q3W for 8 cycles	Nivolumab i.v.	Pembrolizumab 200 mg i.v. Q3W or 400 mg i.v. Q6W for 2 years	CG0070 Inst. + Pembrolizumab 200 mg i.v. Q3W	ALT-803 Inst.	ALT-801 i.v.
**Molecular mechanism of action**	Anti-PD-L1 antibody	Anti-PD-L1 antibody	Anti-PD-1 antibody	Anti-PD-1 antibody	Oncolytic virus + Anti-PD-1 antibody	IL-15RαFc superagonist	T-cell receptor -interleukin-2 fusion molecule
**In combination with**	EBRT-B with 60–66 Gray in 30–33 fractions	Mono or + BGC Inst. or + EBRT-B	Mono or + BGC Inst. or + Linrodostat (BMS-986205) * or + both	BCG Inst.	-	BGC Inst.	Gemcitabine i.v.
**Treatment arms**	1	3	4	2 ♦	1	1	1
**Study phase**	2	1/2	2	3	2	2/3	1b/2
**Histology**	CIS or TaT1/HG or both	CIS or TaT1/HG or both	CIS+/−TaT1/HG	CIS or TaT1/HG or both	CIS+/−pTa/T1 HG	CIS+/−TaT1/HG	CIS or TaT1/HG or both
**Trial status**	NYR	R	ANR	R	R	R	ANR

## Abbreviations

(m)UC	(Metastatic) urothelial carcinoma
(N)MIBC	(Non-)muscle-invasive bladder cancer
AEs	Adverse events
≥G3 AE	≥Grade 3 adverse event
irAE	Immune-related adverse event
sAE	Serious adverse event
APC	Antigen-presenting cells
ASCO-GU	Genitourinary Cancers Symposium of the American Society of Clinical Oncology
AUA	American Urologic Association
BC	Bladder cancer
BCG	Bacille Calmette–Guérin
BPS	Bladder-preserving strategies
CEA	Carcinoembryonic antigen
CIS	Carcinoma in situ
ClTr	Clinical trials
CPI	Checkpoint inhibition
CPS	Combined positive score
CR	Complete response
CT	Center of the tumor
CUETO	Club Urologico Español deTratamiento Oncologico
DDM	Dodecyl maltoside
DoR	Duration of response
EAU	European Association of Urology
EBRT	External Beam Radiotherapy of the Bladder
ECOG	Eastern Cooperative Oncology Group
EFS	Event-free survival
EORTC	European Organisation for Research and Treatment of Cancer
FDA	United States Food and Drug Administration
FoxP3	Forkhead box protein P3
FU	Follow up
G1–3	Grading 1–3
LG	Low grade
HG	High grade
GM-CSF	Granulocyte-macrophage colony-stimulating factor
HG-RFS	High-grade recurrence-free survival
I	Immunoscore
i.v.	Intravenously
iCT	Intravesical chemotherapy
IFN	Interferon
IL	Interleukin
IM	Invasive margin
Maint.	Maintenance
MDSC	Myeloid-derived suppressor cells
MHC	Major histocompatibility complex
MMC	Mitomycin C
MUC	Antigens epithelial mucin 1
n.a.	Not applicable
NA	Not available
NKT-cell	Natural killer T-cell
OM	Oportuzumab monatox
PD	Progressive disease
PD-(L)1	Programmed cell death (ligand)-1
PRISMA	Preferred Reporting Items for Systematic Review and Meta-Analysis statement
Rb	Retinoblastom
rBCG	Repeat BCG
RC	Radical cystectomy
RCT	Randomized controlled trial
RECIST	Response evaluation criteria in solid tumors
RR	Risk ratio
SD	Stable disease
TAM	Tumor-associated macrophage
TIL	Tumor-infiltrating lymphocyte
TLR	Toll-like receptor
TMB	Tumor mutational burden
TU	Tumor
TURBT	Transurethral resection of bladder tumor
UTI	Urinary tract infection
Vp	Viral particles
WHO	World Health Organization
